# Synthesis of Bisimidazole Derivatives for Selective Sensing of Fluoride Ion

**DOI:** 10.3390/molecules22091519

**Published:** 2017-09-11

**Authors:** Liang Zhang, Fang Liu

**Affiliations:** School of Material Science and Engineering, Yancheng Institute of Technology, Yancheng 224051, Jiangsu, China; lf@ycit.cn

**Keywords:** imidazole, fluorescene, phenanthroline, fluoride ion, anion sensor

## Abstract

Rapid and efficient analysis of fluoride ion is crucial to providing key information for fluoride ion hazard assessment and pollution management. In this study, we synthesized one symmetrical structure called 1,4-bis(4,5-diphenyl-1*H*-imidazol-2-yl)benzene (**1a**) and two asymmetrical structures, namely 2-(4-(4,5-diphenyl-1*H*-imidazol-2-yl)phenyl)-1*H*-phenanthro(9,10-*d*)imidazole (**1b**) and 2-(4-(4,5-diphenyl-1*H*-imidazol-2-yl)phenyl)-1*H*-imidazo(4,5-*f*)(1,10)phenanthroline (**1c**), which served as an efficient anion sensor for fluoride ion over a wide range of other anions (Cl^−^, Br^−^, I^−^, NO_3_^−^, ClO_4_^−^, HSO_4_^−^, BF_4_^−^, and PF_6_^−^) owing to imidazole group in the main backbone. The absorption intensity of compound **1a** at λ_max_ 358 nm slightly decreased; however, a new band at λ_max_ 414 nm appeared upon the addition of fluoride ion, while no evident change occurred upon the addition of eight other anions. The photoluminescence intensity of compound **1a** at λ_max_ 426 nm was nearly quenched and fluorescence emission spectra were broadened when fluoride ion was added into dimethyl sulfoxide (DMSO) solution of compound **1a**. Compared with the optical behaviors of the DMSO solution of compound **1a** in the presence of Bu_4_N^+^F^−^, compounds **1b** and **1c** exhibited considerable sensitivity to fluoride ion due to the increase in coplanarity. Furthermore, compared with the fluorescence emission behaviors of the DMSO solutions of compounds **1a** and **1b** in the presence of Bu_4_N^+^F^−^, compound **1c** exhibited the most significant sensitivity to fluoride ion due to the charge transfer enhancement. Consequently, the detection limits of compounds **1a**–**1c** increased from 5.47 × 10^−6^ M to 4.21 × 10^−6^ M to 9.12 × 10^−7^ M. Furthermore, the largest red shift (75 nm) of the DMSO solution compound **1c** in the presence of fluoride ion can be observed. Our results suggest that the increase in coplanarity and the introduction of electron-withdrawing groups to the imidazole backbone can improve the performance in detecting fluoride ion.

## 1. Introduction

The field of anion recognition and sensing has attracted considerable attention in the past decades because different anions play different functions in biological and environmental processes and either inadequate or excessive anions would be harmful [1,2,3,4,5,6,7,8,9,10,11,12]. Among all anions, fluoride ion has been extensively studied because it plays a vital role in medicine, biology, and environmental sciences [13,14,15,16,17,18,19,20,21,22,23,24,25]. Therefore, an efficient analysis of fluoride ion is crucial to providing key information for fluoride ion hazard assessment and pollution management. Many groups have made substantial efforts to design and synthesize optical sensors of fluoride ion in recent years [26,27,28,29,30,31,32]. Among these optical sensors, imidazole-based optical sensors have been extensively investigated because these sensors are easily obtained and exhibit distinctive fluorescence property and strong interaction between N–H fragment and fluoride ion [33,34,35,36,37,38,39,40].

In this study, we synthesized one symmetrical structure called 1,4-bis(4,5-diphenyl-1*H*-imidazol-2-yl)benzene (**1a**) and two asymmetrical structures, namely 2-(4-(4,5-diphenyl-1*H*-imidazol-2-yl)phenyl)-1*H*-phenanthro(9,10-*d*)imidazole (**1b**) and 2-(4-(4,5-diphenyl-1*H*-imidazol-2-yl)phenyl)-1*H*-imidazo(4,5-*f*)(1,10)phenanthroline (**1c**), which have been further used as optical sensors for fluoride ion. The relationship between molecular structures and optical properties for the analysis of fluoride ion has been investigated in terms of five aspects: (1) Compared with mono-imidazole derivatives, the fluorescence quantum yields of bisimidazole are higher; (2) In view of the influence of molecular rigidity on optical performance for the analysis of fluoride ion, two adjacent phenyl rings are connected through C–C bond to increase molecular coplanarity; (3) The introduction of two sp^2^-hybridized *N* atoms as electron-withdrawing substituents into the molecular backbone is intended for the investigation of the electronic effects of substituents; (4) As shown in Scheme S2 (Supplementary Materials), compared with compound **1a**, compound **1b** exhibits better coplanarity which is more favorable for carrier transport and then improves the detection limit of sensors. Phenanthrene group was used as an electron donor in compound **1b** and then charge transfer can easily occur from phenanthrene group (donor) to imidazole group (acceptor). However, when phenanthrene group is changed to 1,10-phenanthroline group, charge transfer cannot easily occur from 1,10-phenanthroline group to imidazole group because 1,10-phenanthroline group is an stronger electron accepor, compared with imidazole group; (5) When compounds **1a**–**1c** dimethyl sulfoxide (DMSO) solution are added with excess fluoride ion, deprotonation of imidazole group occur which changes imidazole group as an electron donor to as an electron acceptor [33,41], leading to different charge transfer process of compounds **1b** and **1c**. Thus, the optical properties of compounds **1a**–**1c** DMSO solution for the analysis of fluoride ion influenced by the molecular structures and electronic properties of substitute groups are investigated in detail.

## 2. Results and Discussion

### 2.1. Synthesis of Compounds ***1a***–***1c***

**Scheme 1:**
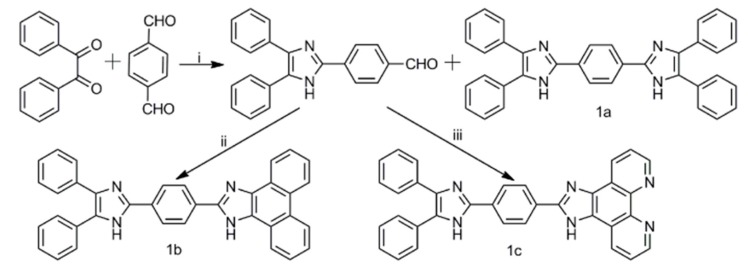
Synthetic route of compounds **1a**–**1c**. (**i**) ammonium acetate, glacial acetic acid; (**ii**) ammonium acetate, glacial acetic acid, phenanthrene-9,10-dione; (**iii**) ammonium acetate, glacial acetic acid, 1,10-phenanthroline-5,6-dione.

Scheme 1 depicts the synthetic procedure for the preparation of compounds **1a**–**1c** according to previous studies [42,43]. The two adjacent phenyl rings of compound **1a** were connected through C–C bond to form compound **1b**, which exhibited a rigid structure and high degree of coplanarity, to investigate the relationship between molecular structures and optical behaviors. Furthermore, two sp^2^-hybridized *N* atoms as electron-withdrawing substituents were introduced into compound **1b** to form compound **1c** to study the electronic effects of substituents. Consequently, compound **1a** displayed a symmetrical structure, whereas compounds **1b** and **1c** had an asymmetrical structure.

### 2.2. Optical Properties of Compounds ***1a***–***1c***

**Figure 1:**
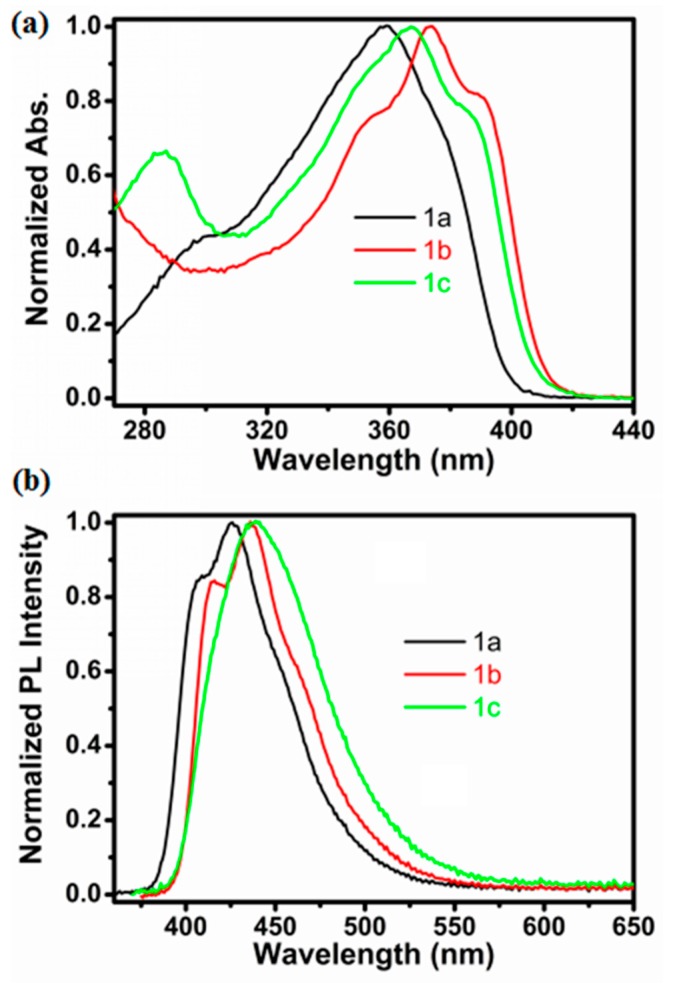
(**a**) Normalized absorption and (**b**) emission spectra of compounds **1a**–**1c** in DMSO solution (λ_ex_ = λ_abs_, 10^−5^ mol L^−^^1^).

Figure 1a illustrates the normalized absorption spectra of compounds **1a**–**1c** in DMSO solution. The absorption spectrum of compound **1a** exhibits two prominent bands at λ_max_ 296 nm and 358 nm, which can be ascribed to a localized aromatic π-π* transition and the absorption of the entire molecule, respectively [3,33]. Compared with the absorption spectrum of compound **1a**, the long peak of compound **1b** is red shifted from 358 nm to 372 nm with two shoulder peaks at λ_max_ 352 nm and 390 nm because the structure of compound **1b** is more coplanar than that of compound **1a** due to the inhibition of C–C single bond rotation of compound **1b**. Although compound **1c** possesses two sp^2^-hybridized *N* atoms as electron-withdrawing substituents, the long peak of compound **1c** is slightly blue shifted from 372 nm (the absorption peak of compound **1b**) to 367 nm with a shoulder peak at λ_max_ 385 nm. This phenomenon is similar to that presented in a previous report regarding symmetrical bisimidazole systems [44]. Molar absorption coefficients of compounds **1a**–**1c** are 6.0 × 10^4^, 5.2 × 10^4^, and 5.2 × 10^4^ L mol^−^^1^ cm^−^^1^, respectively. Compounds **1a**–**1c** emit a strong blue light in the DMSO solution, and corresponding emission spectra are shown in Figure 1b. As shown in Figure 1b, two emission bands exist at λ_em_ = 406 nm and λ_em_ = 426 nm under excitation at λ_ex_ 358 nm for compound **1a**, λ_em_ = 416 nm and λ_em_ = 436 nm under excitation at λ_ex_ 372 nm for compound **1b**, and λ_em_ = 438 nm under excitation at λ_ex_ 367 nm for compound **1c**. The long emission wavelength is red shifted from compound **1a** to compound **1b** to compound **1c** due to the increase in coplanarity, which can lead to the expansion of the π electron delocalization.

**Figure 2:**
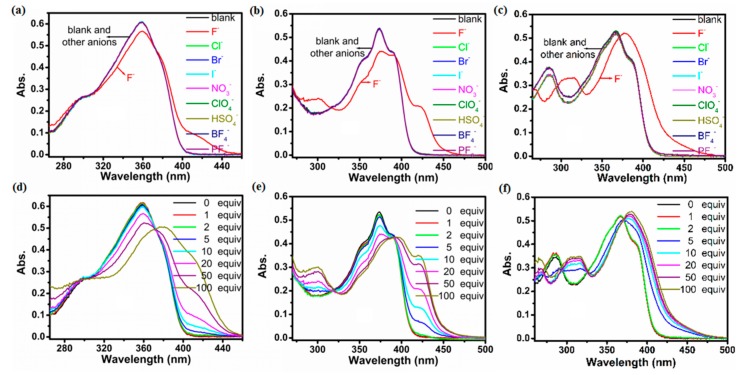
UV/Vis absorption spectra of DMSO solutions of (**a**) compound **1a**; (**b**) compound **1b**; and (**c**) compound **1c** (10^−^^5^ mol L^−^^1^) in the absence (blank) and presence of nine anions (20 equiv.). UV/Vis absorption spectra of DMSO solutions of (**d**) compound **1a**; (**e**) compound **1b**; and (**f**) compound **1c** (10^−^^5^ mol L^−^^1^) with different amounts of Bu_4_N^+^F^−^.

**Scheme 2:**
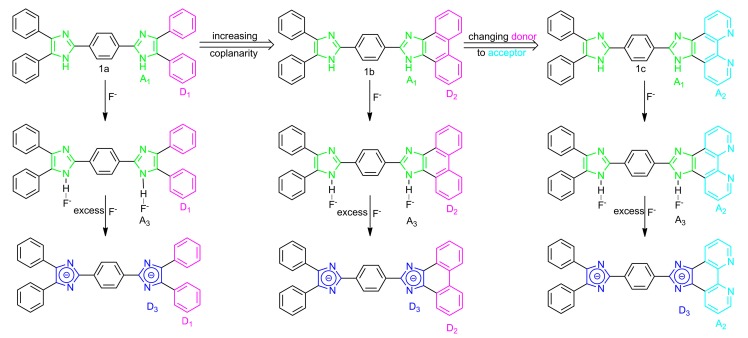
Schematic diagram for electron transfer process of compounds **1a**–**1c** in the presence of Bu_4_N^+^F^−^ (A = electron acceptor; D = electron donor).

Although imidazole derivatives have been used as chemosensors for fluoride ion, systematic studies on the effects of coplanarity and electron-withdrawing substituents have rarely been reported. Figure 2a exhibits the absorption spectra of the DMSO solution of compound **1a** in the presence of nine anions (F^−^, Cl^−^, Br^−^, I^−^, NO_3_^−^, ClO_4_^−^, HSO_4_^−^, BF_4_^−^, and PF_6_^−^, 20 equiv.). The absorption intensity at λ_max_ 358 nm slightly decreases, but a new band at λ_max_ 414 nm appears, while no evident change occurred upon the addition of eight other anions. The new band at λ_max_ 414 nm is attributed to the variations of the electronic transition due to the occurrence of a strong interaction between fluoride ion and N–H groups. As shown in Scheme 2, the imidazole group serves as an electron acceptor before the addition of Bu_4_N^+^F^−^, but the imidazole group acts as an electron donor after the addition of Bu_4_N^+^F^−^, which leads to the charge transfer enhancement. The titration absorption spectra of the DMSO solution of compound **1a** with the addition of different amounts of Bu_4_N^+^F^−^ have been examined to verify this phenomenon, as shown in Figure 2d. The absorption intensity at λ_max_ 358 nm decreases, and the absorption intensity at λ_max_ 414 nm steadily increases with the increase in the amount of Bu_4_N^+^F^−^. Meanwhile, the absorption peak at λ_max_ 358 nm is red shifted to 378 nm when 100 equiv. Bu_4_N^+^F^−^ is added into the DMSO solution of compound **1a**, suggesting that compound **1a** can be a candidate as a chemosensor for fluoride ion. The absorption behavior of the DMSO solution of compound **1b** in the presence of Bu_4_N^+^F^−^ is similar to that of the DMSO solution of compound **1a**. However, a new band at λ_max_ 420 nm can be easily observed. Compared with the absorption behaviors of the DMSO solutions of compounds **1a** and **1b** in the presence of Bu_4_N^+^F^−^, the absorption behavior of the DMSO solution of compound **1c** is different to some extent. The absorption peak at λ_max_ 367 nm is red shifted with the increase in the amount of Bu_4_N^+^F^−^.

**Figure 3:**
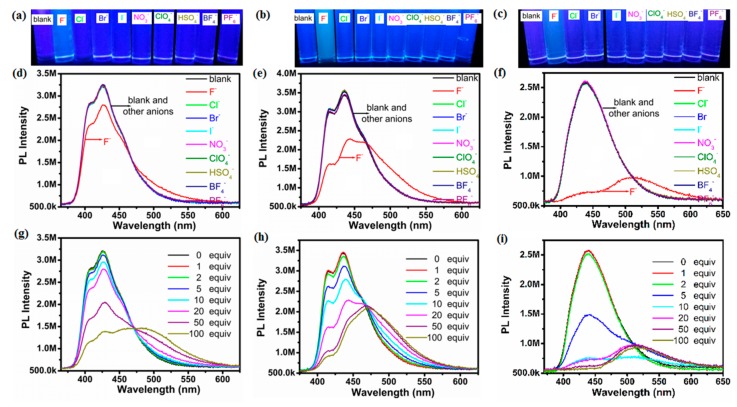
Photographs of the DMSO solutions of (**a**) compound **1a**; (**b**) compound **1b**; and (**c**) compound **1c** (10^−^^5^ mol L^−^^1^) in the absence (blank) and presence of nine anions (20 equiv.) taken under UV light at 365 nm. Emission spectra of DMSO solutions of (**d**) compound **1a**; (**e**) compound **1b**, and (**f**) compound **1c** (10^−^^5^ mol L^−^^1^) in the absence (blank) and presence of nine anions (20 equiv.). UV/Vis absorption spectra of DMSO solutions of (**g**) compound **1a**; (**h**) compound **1b**; and (**i**) compound **1c** (10^−^^5^ mol L^−^^1^) with different amounts of Bu_4_N^+^F^−^.

The fluorescence emission behaviors of the DMSO solutions of compounds **1a**–**1c** in the presence of nine anions (F^−^, Cl^−^, Br^−^, I^−^, NO_3_^−^, ClO_4_^−^, HSO_4_^−^, BF_4_^−^, and PF_6_^−^, 20 equiv.) are also investigated, and the corresponding emission spectra are shown in Figure 3. As shown in Figure 3a,d, the photoluminescence (PL) intensity of compound **1a** is slightly quenched, and the fluorescence emission spectra are slightly broadened in the presence of fluoride ion (20 equiv.), while no evident fluorescence change occurred in the presence of eight other anions. Figure 3g depicts the titration emission spectra of the DMSO solution of compound **1a** with the addition of different amounts of Bu_4_N^+^F^−^. The PL intensity at λ_max_ 426 nm is nearly quenched, and the fluorescence emission spectra are further broadened when 100 equiv. Bu_4_N^+^F^−^ are added into the DMSO solution of compound **1a**. The UV/Vis absorption and fluorescence emission spectra of compound **1a** shows a fact that firstly formation of N-H^…^F hydrogen bond and subsequent deprotonation with adding excess Bu_4_N^+^F^−^ is responsible for the behaviour of compound **1a** (Scheme 2). Figure S11 shows that the linear regression equation of compound **1a** was y = 4.781 − 0.00347x, and the slope was −0.00347 (Supplementary Materials). The detection limit of compound **1a** was calculated to be 5.47 × 10^−6^ M with the equation: detection limit = 3S_d_/ρ, where S_d_ is the standard deviation of blank measurement, and ρ is the slope between the fluorescence intensity versus fluoride ion concentration [45,46]. The emission behavior of the DMSO solution of compound **1b** in the presence of Bu_4_N^+^F^−^ is similar to that of the DMSO solution of compound **1a**. However, the PL intensity of compound **1b** is quenched, and a new band at λ_max_ 470 nm appears in the presence of fluoride ion (20 equiv.). The detection limit of compound **1b** was calculated to be 4.21 × 10^−6^ M according to Figure S12. Compared with the emission behaviors of the DMSO solutions of compounds **1a** and **1b** in the presence of Bu_4_N^+^F^−^, the emission behavior of the DMSO solution of compound **1c** is different. The PL intensity of compound **1c** at λ_max_ 438 nm is dramatically quenched, and a new band at λ_max_ 513 nm is easily observed in the presence of fluoride ion (20 equiv.). Furthermore, the PL peak of compound **1c** at λ_max_ 438 nm disappears when 50 equiv. Bu_4_N^+^F^−^ are added into the DMSO solution of compound **1c**. The detection limit of compound **1c** was calculated to be 9.12 × 10^−7^ M according to Figure S13. As shown in Scheme 2, the imidazole group acts as as an electron acceptor before the addition of Bu_4_N^+^F^−^, charge transfer can easily occur from diphenyl (for compound **1a**) or phenanthrene (for compound **1b**) group (donor) to imidazole group (acceptor) but charge transfer can not easily occur from 1,10-phenanthroline group (for compound **1c**) to imidazole group because 1,10-phenanthroline group is an stronger electron accepor. While the imidazole group acts an electron donor after the addition of excess Bu_4_N^+^F^−^, which leads to the charge transfer enhancement from the imidazole group to 1,10-phenanthroline. Therefore, the largest red shift (75 nm) of the DMSO solution of compound **1c** is in the presence of Bu_4_N^+^F^−^.

## 3. Conclusions

In summary, one symmetrical structure (**1a**) and two asymmetric structures (**1b** and **1c**) were successfully synthesized. Interestingly, compounds **1a**–**1c** can act as efficient anion sensors for fluoride ion over a wide range of other anions (Cl^−^, Br^−^, I^−^, NO_3_^−^, ClO_4_^−^, HSO_4_^−^, BF_4_^−^ and PF_6_^−^) owing to the imidazole group in the main backbone. Compared with the optical behaviors of the DMSO solution of compound **1a** in the presence of Bu_4_N^+^F^−^, compounds **1b** and **1c** exhibit considerable sensitivity to fluoride ion due to the increase in the coplanarity. Furthermore, compared with the fluorescence emission behaviors of the DMSO solutions of compounds **1a** and **1b** in the presence of Bu_4_N^+^F^−^, compound **1c** exhibits the most significant sensitivity to fluoride ion due to charge transfer enhancement. Consequently, the detection limits of compounds **1a**–**1c** increase from 5.47 × 10^−^^6^ M to 4.21 × 10^−^^6^ M to 9.12 × 10^−^^7^ M. Our results suggest that the increase in the coplanarity and the introduction of electron-withdrawing groups to the imidazole backbone can improve the performance in detecting fluoride ion. We will introduce poly(ethylene glycol) methyl ether into start materials to improve the solubily of target compounds in water in our future work.

## Supplementary Materials

Supplementary Materials are available online. Figure S1. ^1^H NMR spectrum of compound 4-(4,5-diphenyl-1*H*-imidazol-2-yl)benzaldehyde in DMSO-*d*_6_. Figure S2. ^1^H NMR spectrum of compound **1a** in DMSO-*d*_6_. Figure S3. ^1^H NMR spectrum of compound **1b** in DMSO-*d*_6_. Figure S4. ^1^H NMR spectrum of compound **1c** in DMSO-*d*_6_. Figure S5. MS spectrum of compound **1a**. Figure S6. MS spectrum of compound **1b**. Figure S7. MS spectrum of compound **1c**. Figure S8. Correlation curves of compound **1a** at 358 nm and 414 nm adding different equivalents of F^−^. Figure S9. Correlation curves of compound **1b** at 372 nm and 420 nm adding different equivalents of F^−^. Figure S10. Correlation curves of compound **1c** at 367 nm and 380 nm adding different equivalents of F^−^. Figure S11. Change in PL intensity of compound **1a** (10 uM) upon titration with F^−^. Figure S12. Change in PL intensity of compound **1b** (10 uM) upon titration with F^−^. Figure S13. Change in PL intensity of compound **1c** (10 uM) upon titration with F^−^.
