# Supplementary material for: Synthesis of Bisimidazole Derivatives for Selective Sensing of Fluoride Ion
Source: Molecules. 2017 Sep 11;22(9):1519. doi: 10.3390/molecules22091519 (PMC6151418; doi:10.3390/molecules22091519)
Supplement: Supplementary file 1 [file molecules-22-01519-s001.pdf]

Supplementary Information

Synthesis of bisimidazole derivatives for selective sensing of fluoride ion

Liang Zhang,*[,a] and Fang Liu[a]

[a]School of Material Science and Engineering, Yancheng Institute of Technology, Yancheng 224051, Jiangsu, China, Email: zhangliang@ycit.edu.cn

General Information: All the starting materials (analytic pure) were purchased from either TCI or Sinopharm Chemical Reagent Co, Ltd and were used as received without further purification.

Using DMSO-d_6 as solvent and tetramethylsilane (TMS) as the internal standard, ^{1}H NMR NMR spectra were measured on INOVA 400 MHz NMR spectrometer at ambient temperature. UV-vis absorption spectra were determined on a Shimadzu RF540 spectrophotometer.

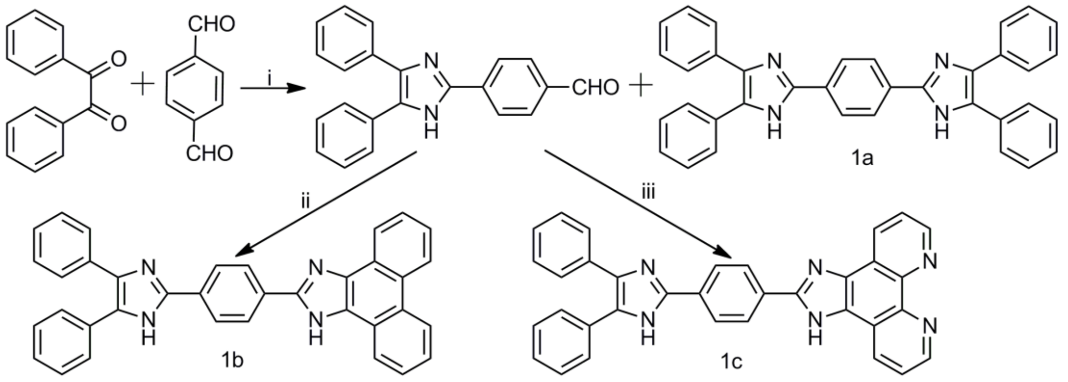

Scheme S1. Synthetic routes for the preparation of three compounds 1a-1c: (i) ammonium acetate, glacial acetic acid; (ii) ammonium acetate, glacial acetic acid, phenanthrene-9,10-dione; (iii) ammonium acetate, glacial acetic acid, 1,10-phenanthroline-5,6-dione.

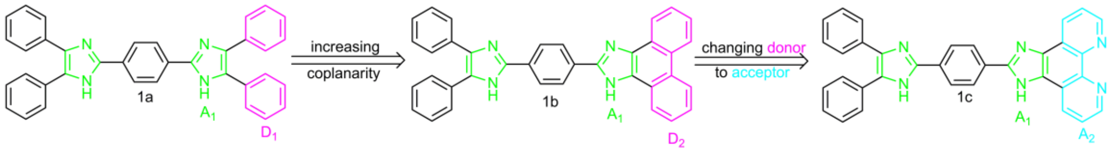

Scheme S2. Structural changes of compounds 1a-1c.

Experimental Section

Synthesis of 4-(4,5-diphenyl-1*H*-imidazol-2-yl)benzaldehyde

A mixture of bezil (10 mmol,2.10 g), terephthalaldehyde (12 mmol, 1.61 g), ammonium acetate (100 mmol,7.70 g) and glacial acetic acid(30 mL) was heated at 120 ^{o}C for 3h and monitored by TLC. The mixture solution was poured into ice-water and adjusted pH = 7 with aqueous ammonia . The precipitate was collected on a filter and washed with cold water, dried, and then separated by column chromatography with hexane/ethyl acetate (hexane/ethyl acetate, V:V=3:1) as eluent. The green solid (4.6 mmol, 1.47 g, 46%) was obtained after evaporation of solvents.

^{1}H NMR (400 MHz, DMSO-d_6) δ 13.02 (s, 1H), 10.03 (s, 1H), 8.30 (d, *J* = 8.3 Hz, 2H), 8.02 (d, *J* = 8.3 Hz, 2H), 7.54 (dd, *J* = 13.8, 7.1 Hz, 4H), 7.47 (t, *J* = 7.3 Hz, 2H), 7.43 – 7.38 (m, 1H), 7.32 (t, *J* = 7.4 Hz, 2H), 7.26 (d, *J* = 7.3 Hz, 1H).

Synthesis of 1,4-bis(4,5-diphenyl-1*H*-imidazol-2-yl)benzene (1a)

A mixture of bezil (15 mmol,3.15 g), terephthalaldehyde (5 mmol ,0.67 g) ,ammonium acetate(100 mmol, 7.7 g) and glacial acetic acid (50 mL) was heated at 120 ^{o}C for 24h. The reaction mixture was poured into ice-water and adjusted pH = 7 with aqueous ammonia. The precipitate was collected on a filter and washed with cold water, dried vaccum. The light yellow solid (4.05 mmol, 2.07 g, 81%) was obtained by crystallization from glacial acetic acid.

^{1}H NMR (400 MHz, DMSO-d_6) δ 12.77 (s, 2H), 8.19 (s, 4H), 7.55 (s, 8H), 7.39 (m, 12H).

Ion Formula, $C_{36}H_{25}N_4$, Calc. Mass 513.2072, Found 513.2086.

Synthesis of 2-(4-(4,5-diphenyl-1*H*-imidazol-2-yl)phenyl)-1*H*-phenanthro[9,10-*d*]imidazole (1b)

A mixture of 1,10-phenanthroline-5,6-dione (1 mmol, 0.21 g), 4-(4,5-diphenyl-1*H*-imidazol-2-yl)benzaldehyde (1 mmol,0.324g), ammonium acetate (20 mmol, 1.54 g) and glacial acetic acid (50 mL) was heated at 120 ^{o}C for 24 h and then cooled to room temperature. The reaction mixture was poured into ice-water and adjusted pH = 7 with aqueous ammonia. The yellow solid (0.91 mmol, 0.47 g, 91%) was collected on a filter washed with hot ethanol and glacial acetic acid.

^{1}H NMR (400 MHz, DMSO-d_6) δ 13.52 (s, 1H), 12.87 (s, 1H), 8.89 (dd, *J* = 14.1, 8.3 Hz, 2H), 8.61 (dd, *J* = 16.3, 7.8 Hz, 2H), 8.43 (d, *J* = 8.5 Hz, 2H), 8.32 (d, *J* = 8.5 Hz, 2H), 7.76 (dd, *J* = 13.8, 7.2 Hz, 2H), 7.71 – 7.63 (m, 2H), 7.58 (dd, *J* = 17.5, 7.4 Hz, 4H), 7.49 (t, *J* = 7.3 Hz, 2H), 7.36 (dd, *J* = 21.3, 14.0 Hz, 4H).

Ion Formula, $C_{36}H_{27}N_4$, Calc. Mass 515.2230, Found 515.2230.

Synthesis of 2-(4-(4,5-diphenyl-1H-imidazol-2-yl)phenyl)-1*H*-imidazo[4,5-f][1,10]phenanthroline (1c)

The preparation of compound 1c is similar to that of compound 1b from 1,10-phenanthroline-5,6-dione with yield 83%.

^{1}H NMR (400 MHz, DMSO-d_6) δ 9.39 (s, 2H), 9.11 (s, 2H), 8.52 (d, *J* = 7.8 Hz, 2H), 8.33 (d, *J* = 7.9 Hz, 2H), 8.13 (d, *J* = 4.6 Hz, 2H), 7.56 (d, *J* = 7.1 Hz, 4H), 7.40 (dd, *J* = 14.4, 7.0 Hz, 6H).

Ion Formula, $C_{34}H_{23}N_6$, Calc. Mass 515.1979, Found 515.1957.

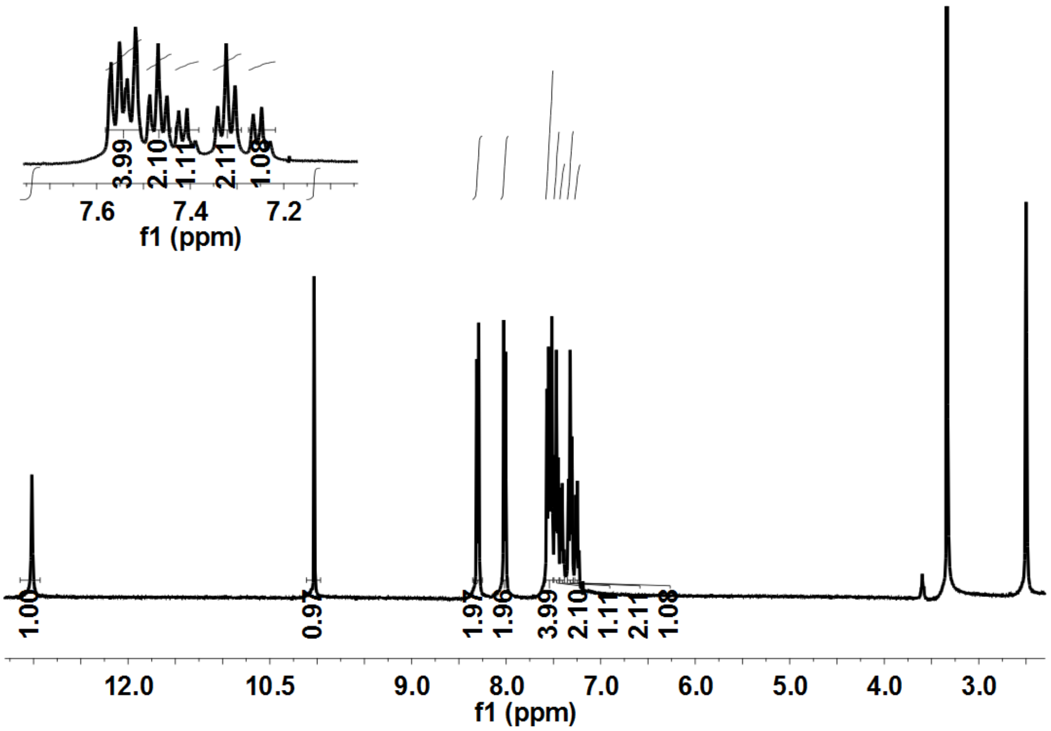

Figure S1. ^{1}H NMR spectrum of compound 4-(4,5-diphenyl-1*H*-imidazol-2-yl)benzaldehyde in DMSO-d_6.

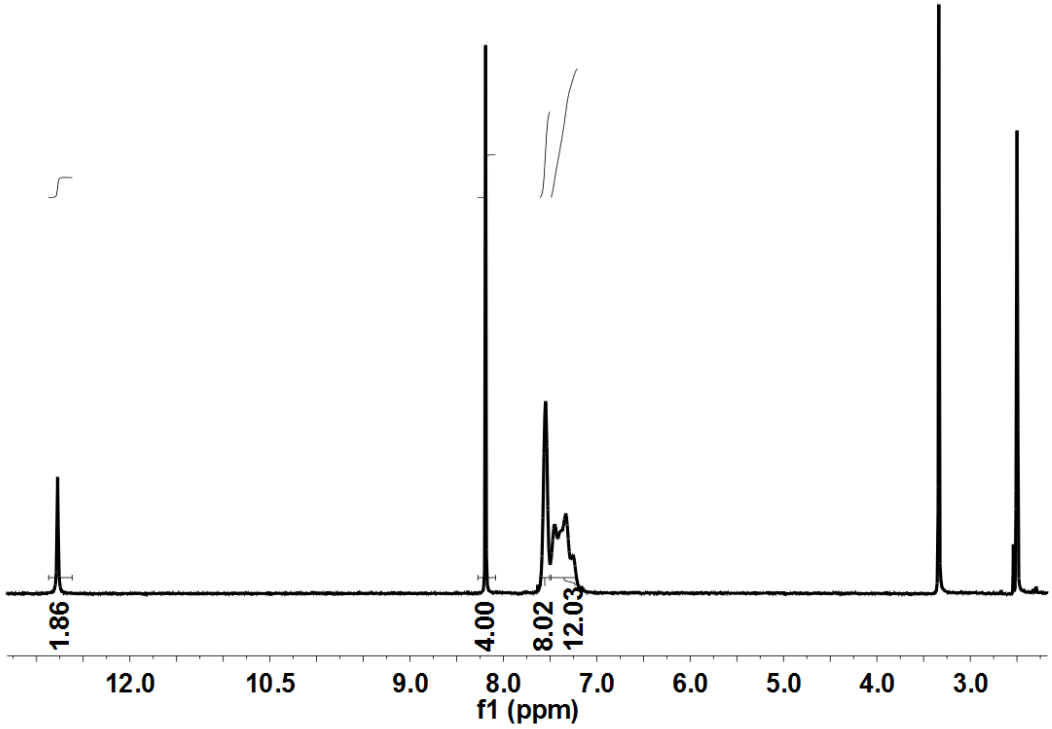

Figure S2. ^{1}H NMR spectrum of compound 1a in DMSO-d_6.

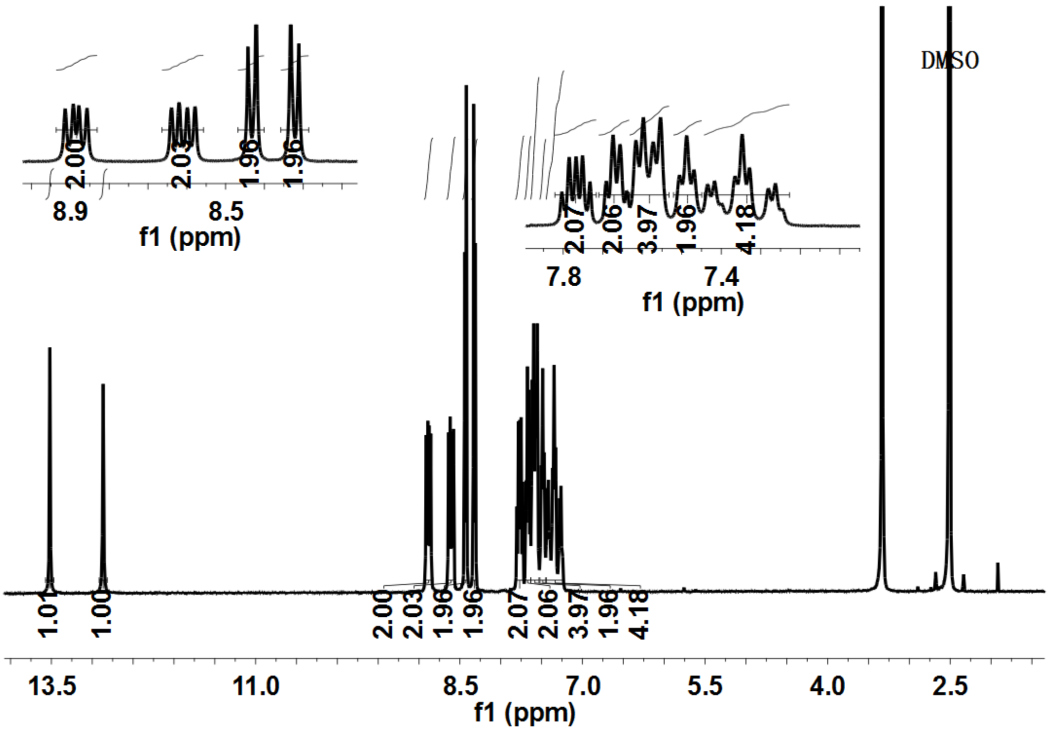

Figure S3. ^{1}H NMR spectrum of compound 1b in DMSO-d_6.

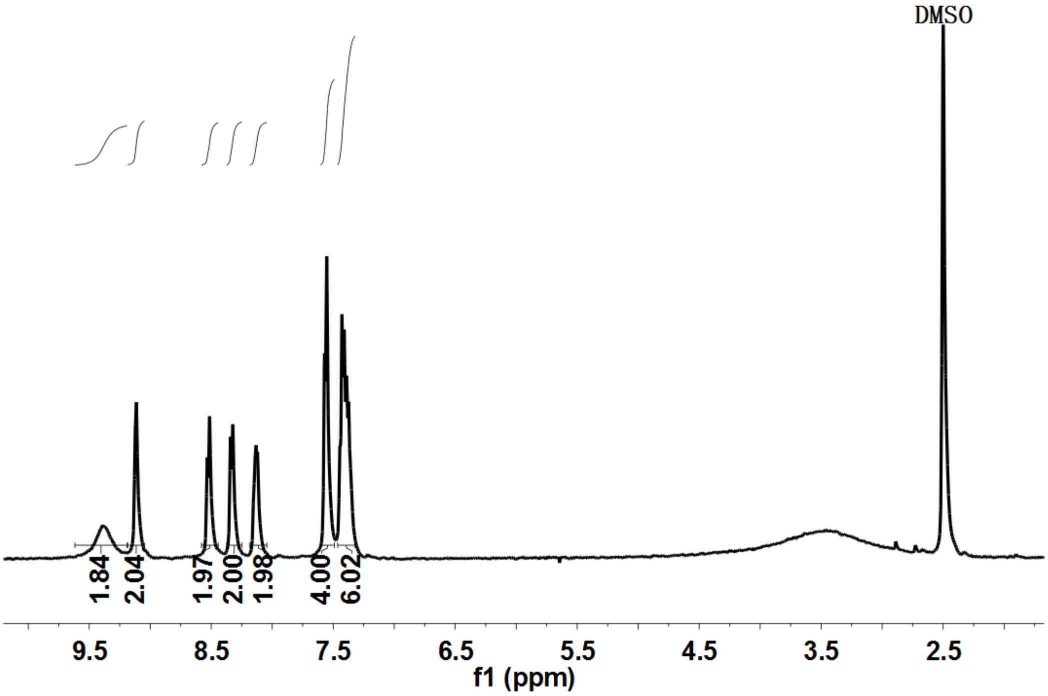

Figure S4. ^{1}H NMR spectrum of compound 1c in DMSO-d_6.

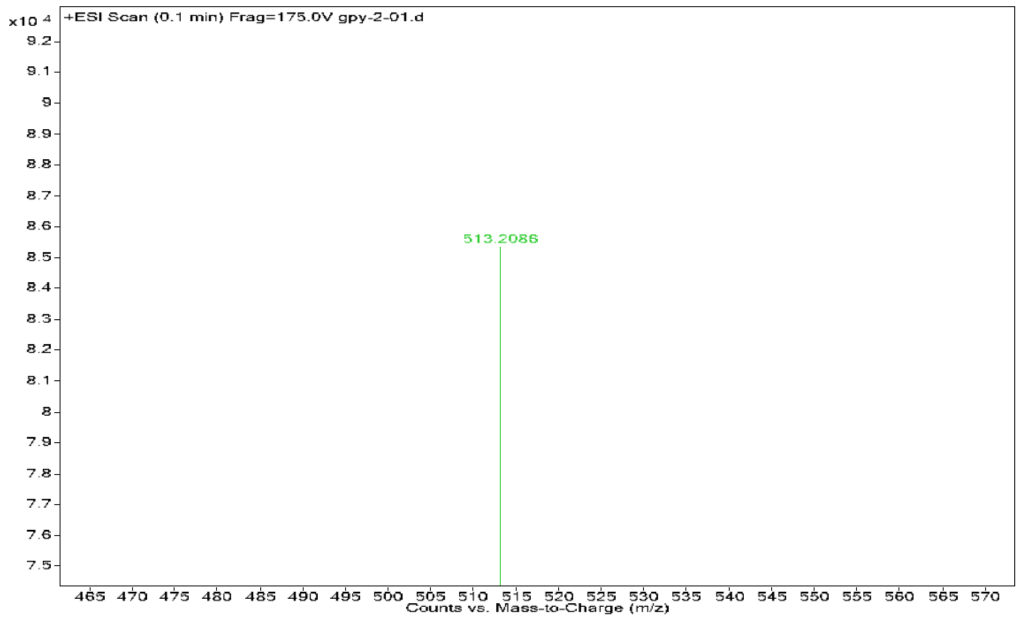

Figure S5. MS spectrum of compound 1a.

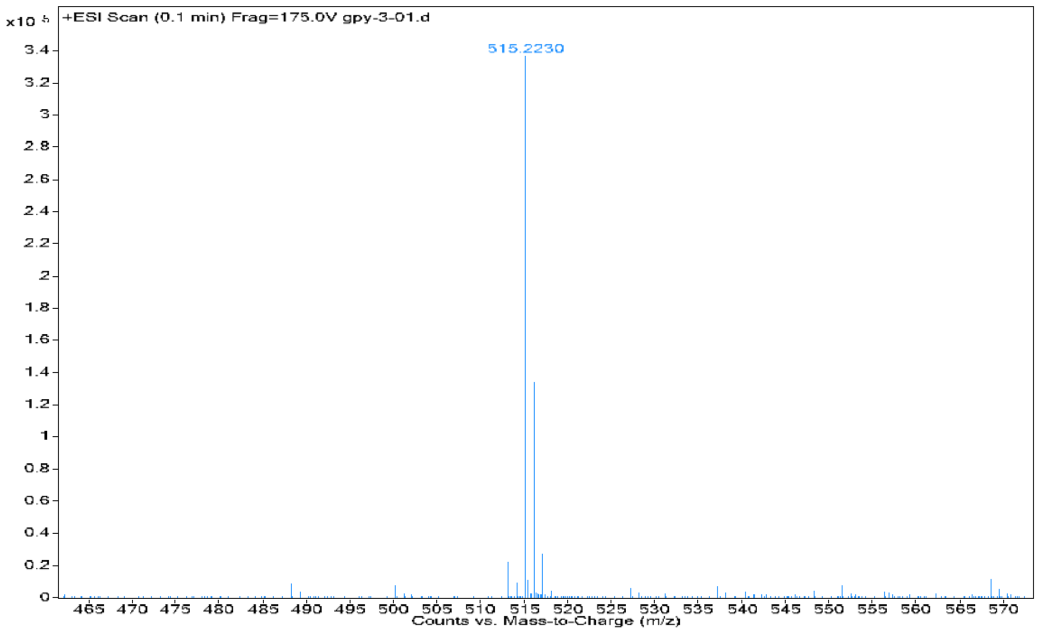

Figure S6. MS spectrum of compound 1b.

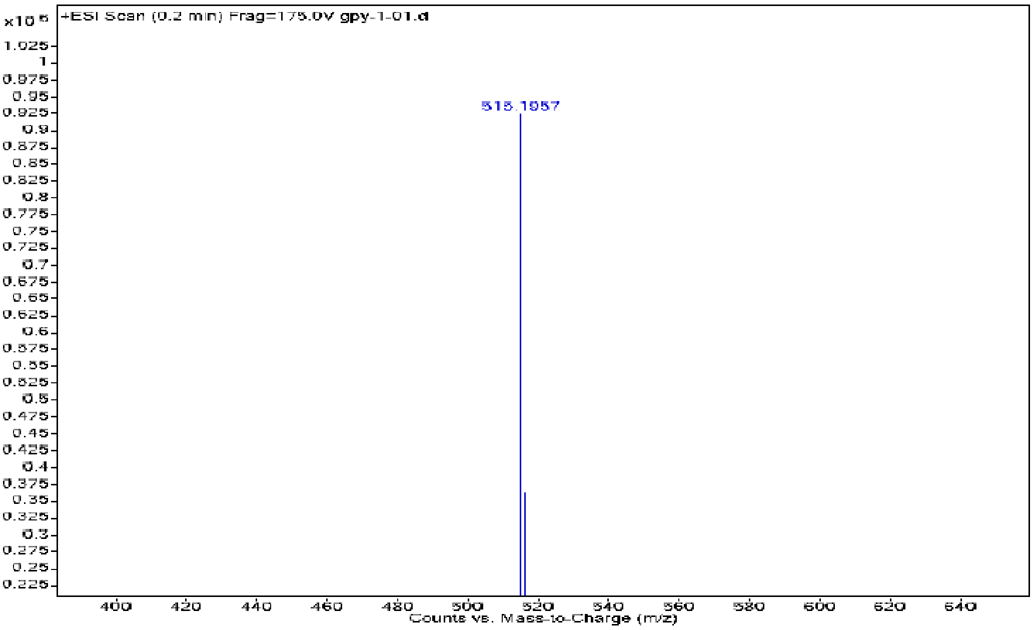

Figure S7. MS spectrum of compound 1c.

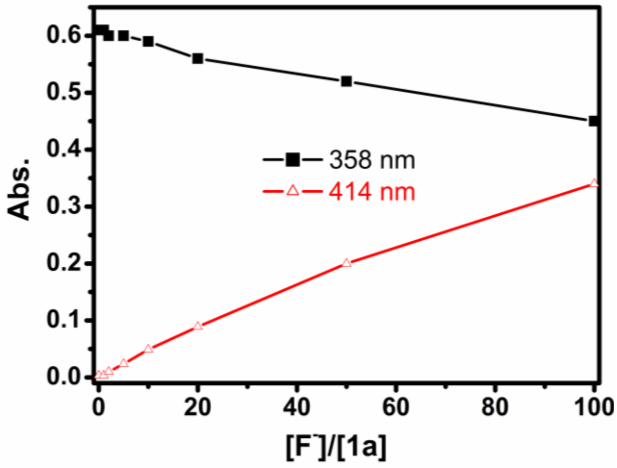

Figure S8. Correlation curves of compound 1a at 358 nm and 414 nm adding different equivalents of F^-.

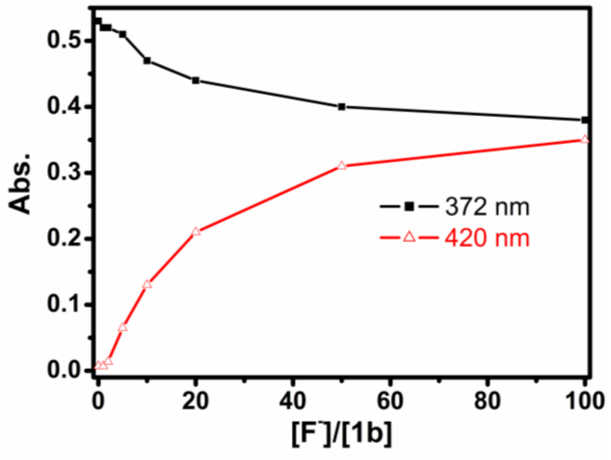

Figure S9. Correlation curves of compound 1b at 372 nm and 420 nm adding different equivalents of F^-.

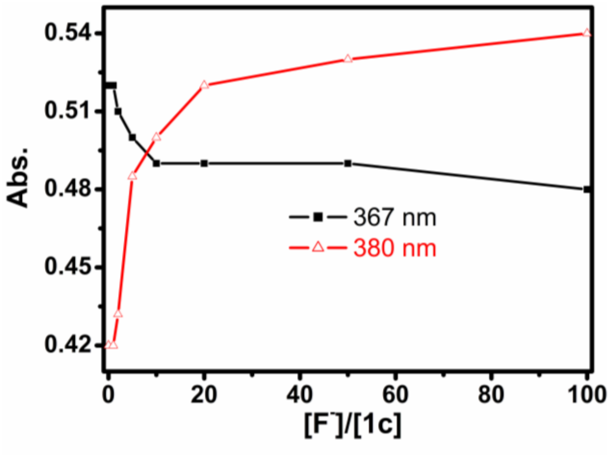

Figure S10. Correlation curves of compound 1c at 367 nm and 380 nm adding different equivalents of F^-.

Calculation of detection limit: The detection limit of compounds 1a-1c for F^- were obtained from fluorescence titration. The compounds 1a-1c (10^{-5} mol L^{-1}) in DMSO solution were treated with various concentrations of F^-, and the relative fluorescence intensity were plotted as a function of the F^- concentration (Figure S11-S13). The detection limit of compounds 1a-1c, based on equation: detection limit = $3S_d/\rho$, where S_d is the standard deviation of blank measurement, and ρ is the slope between the fluorescence intensity versus fluoride ion concentration, were found to be 5.47×10^{-6}, 4.21×10^{-6}, 9.12×10^{-7} mol/L, respectively.

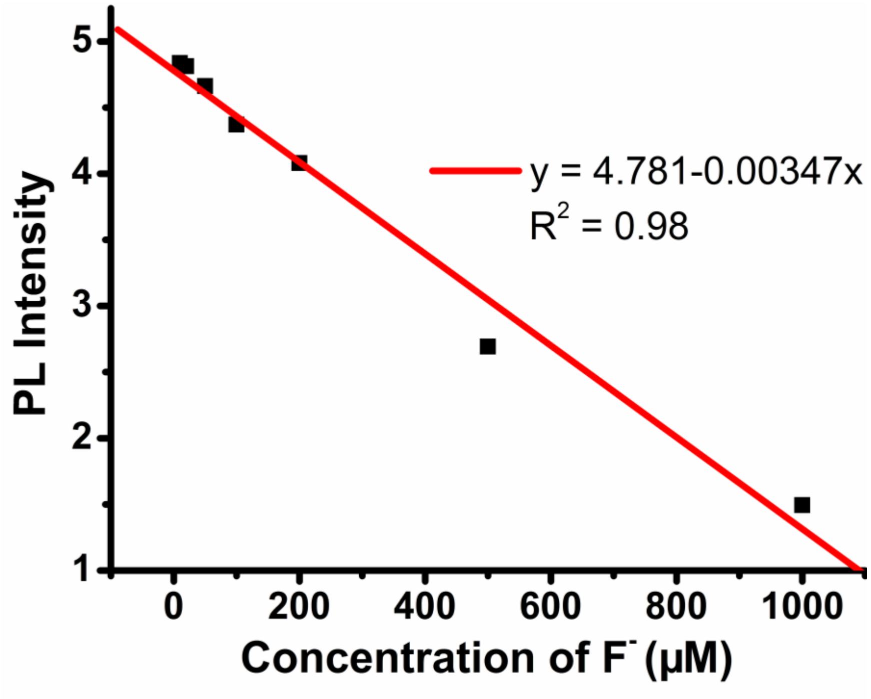

Figure S11. Change in PL intensity of compound 1a (10 uM) upon titration with F^-.

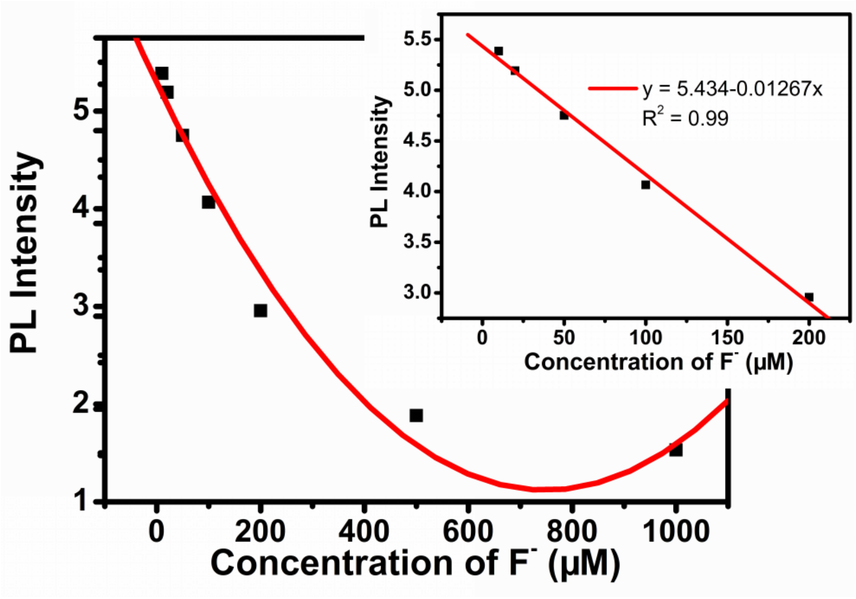

Figure S12. Change in PL intensity of compound 1b (10 uM) upon titration with F^-.

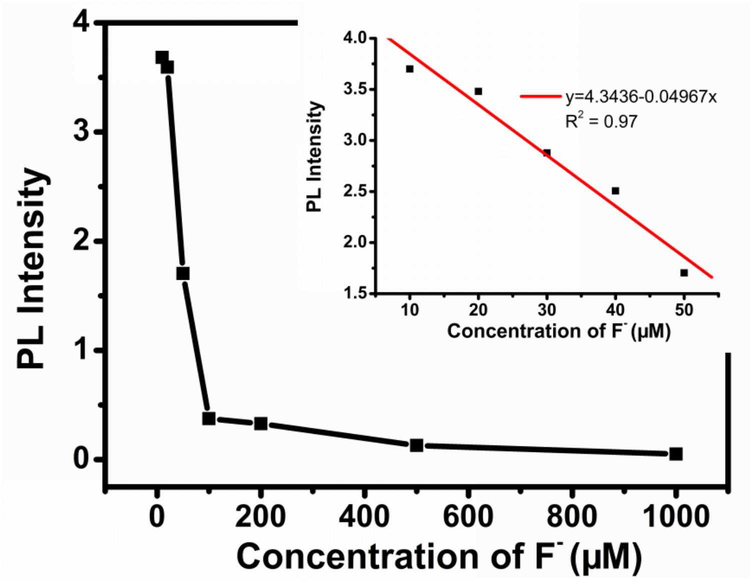

Figure S13. Change in PL intensity of compound 1c (10 uM) upon titration with F^-.
